# Confronting fear and uncertainty: adults’ experiences of undergoing a food challenge test for food allergy

**DOI:** 10.1080/17482631.2026.2626841

**Published:** 2026-02-06

**Authors:** Melina Makatsori, Anne Miles

**Affiliations:** a Specialist Allergy and Clinical Immunology Department, University College London Hospitals NHS Foundation Trust, London, United Kingdom; b Royal Brompton and Harefield NHS Foundation Trust, Sydney Street, London, United Kingdom; c School of Psychological Sciences, Birkbeck, University of London, London, United Kingdom

**Keywords:** Food allergy, qualitative research, food challenge test, psychology, fear, uncertainty

## Abstract

**Background:**

Oral food challenges are indicated when food allergies cannot be confirmed by clinical history or investigations, such as blood or skin-prick tests. However, few studies have examined adults' experiences of oral food challenges.

**Methods:**

Semi-structured, individual interviews were conducted with 18 adults who had undergone an oral food challenge at a UK hospital.

**Results:**

Three main themes were identified using thematic analysis, describing experiences before, during, and after testing: “A limited and scared life,” “facing fear and uncertainty in a safe environment,” and “living with revised boundaries.” Prior to the test, participants described a life characterized by fear of reactions, hypervigilance, planning and restrictive diets, limiting social participation. During testing, participants experienced an emotional rollercoaster, confronting something previously avoided, which could potentially cause a severe reaction. After testing, participants reported reduced fear and uncertainty following clarification of their allergies, leading to greater freedom, and for those who tested negative, a return to viewing food as a source of pleasure, rather than fear.

**Conclusions:**

Oral food challenges reduce fear and uncertainty surrounding allergies, providing clarity about which foods trigger reactions. Greater availability of oral food challenges would enable adults with suspected allergies to live less restricted and more enjoyable lives.

## Introduction

For individuals with food allergies, ingestion of some types of food can lead to potentially life-threatening allergic reactions. Food allergies are an important and emerging health problem with an increasing prevalence in recent decades. Data suggest that more adults than previously acknowledged may be affected, with at least 10.8% affected in the US (Gupta et al., 2019) and prevalence estimates in European adults ranging up to 5.6% in the EuroPrevall population-based study (Lyons et al., 2019).

Food allergies in adults can reflect a persistent allergy from childhood or *de novo* sensitisation to food allergens encountered after childhood and the development of new allergies. There is limited data regarding food allergies beginning in adulthood, but empirical evidence suggests food allergies that start in adulthood often persist (Lee et al., 2024; Sicherer et al., 2020). A large US survey of 40,443 adults found that 48.0% with a reported food allergy developed one or more allergies during adulthood and over a quarter of food-allergic adults reported developing allergies only in adulthood (Gupta et al., 2019).

In evaluating a patient with suspected food allergy, a thorough medical history is very important for identifying symptoms and guiding diagnostic tests (Muraro et al., 2014; Santos et al., 2023). Demonstration of allergen-specific immunoglobulin E (sIgE) detected by skin prick tests or immunoassays of serum sIgE levels aids in the diagnosis of IgE-mediated food allergies (O’Keefe et al., 2014). However, sensitization alone is insufficient to define food allergies (Gupta et al., 2018; Santos et al., 2023). This is because individuals can develop allergic sensitization, as evidenced by the presence of allergen-specific IgE to food allergens, without clinical symptoms upon exposure to these foods. IgE-mediated food allergy therefore requires both the presence of sensitization and the development of specific signs and symptoms upon exposure to that food. When the diagnosis cannot be confirmed based on clinical history and these tests, an oral food challenge is indicated to confirm or exclude allergies.

Oral food challenges are indicated in different scenarios, such as: (a) to confirm the diagnosis of allergy when the cause is uncertain despite skin prick and sIgE testing; (b) to demonstrate tolerance when an allergy is suspected to have been outgrown, or when allergy tests suggest tolerance but the food has never been eaten before and the patient is concerned about introducing it at home; or (c) when assessing tolerance to cross-reactive foods (Muraro et al., 2014; Sampson et al., 2024).

A food challenge involves the graded administration of a potential culprit food. It is performed as a day-case procedure under medical supervision in specialist allergy centres, where individuals are observed for any evidence of a clinical reaction after eating incremental doses of a suspected food according to guidelines (Bindslev-Jensen et al., 2004; Bird et al., 2020; Sampson et al., 2024). There are various types of oral food challenges, and the type of challenge chosen for the assessment of clinical reactivity depends on the potential for bias in the interpretation of results. Double-blind, placebo-controlled food challenge (DBPCFC) is the gold standard and the most rigorous type of challenge (Sampson et al., 2012). Although DBPCFCs can reliably predict clinical reactivity, they are labour- and time-intensive. It is more practical to administer an open food challenge, which is the most commonly used type of challenge in daily clinical practice.

A challenge test is considered negative when no symptoms occur following food consumption according to standard protocols, including observation for at least one hour after the last administered dose, which amounts to the equivalent of a normal portion of the food. A positive outcome requires the presence of symptoms deemed compatible with an allergic response by the investigators and is accompanied by objective physical signs.

The psychological impact of food allergies is different from that of most chronic diseases, with affected individuals having no regular symptoms but living with a constant risk of life-threatening reactions. Food allergy sufferers must always be vigilant, read food labels -which may be time-consuming and frustrating- and live with the possibility of accidental exposure when food ingredients are changed, or when others are unaware of the danger of food allergies. Food allergies have a significant impact on quality of life and are associated with increased anxiety, fear of accidental exposures, and social limitations (DunnGalvin & Hourihane, 2016). Disease-specific quality-of-life instruments, including the Food Allergy Quality of Life Questionnaire-Adult Form and related validated measures, demonstrate reduced quality of life in affected adults, with substantial psychosocial burden related to emotional impact and social restrictions, as well as mental health concerns including anxiety and panic related to the condition (Flokstra-de Blok et al., 2009; Casale et al., 2024; Warren et al., 2021).

Despite food challenges being the gold standard test for food allergy diagnosis, very limited research has been conducted on the experience and impact of undergoing a food challenge test in adults. A systematic review of seven studies found that oral food challenges are associated with improved food allergy-specific health-related quality of life (HRQoL) and reduced parental burden (Kansen et al., 2018). However, the majority of these studies have focused on children, adolescents, or their caregivers, with only one study including adults who had undergone DBPCFCs (Sampson et al., 2012). In the latter study, HRQoL scores improved significantly after DBPCFC when all outcomes of the test were combined compared with a control group. A greater improvement in HRQoL was observed following a negative outcome, with a smaller but still significant improvement after a positive outcome (Sampson et al., 2012). In a more recent study, adults with a negative food challenge outcome had better HRQoL than those with a food allergy confirmed via clinical history, skin prick testing, and/or sIgE testing, or a positive oral food challenge, with no differences observed between the latter two groups (food challenge vs. no challenge) (Makatsori & Miles, 2021).

In terms of qualitative research, parental and adolescent perceptions of food challenges have been examined (Correa et al., 2022; Strinnholm et al., 2010; Strinnholm et al., 2017), but to our knowledge, no qualitative studies have examined both the experience of living with a food allergy and how undergoing an oral food challenge may impact this experience in adults. In order to improve current clinical practice and patient outcomes, information is required about individuals’ views and experiences of having such a diagnostic test. The impact of food challenges on health-related quality of life has already been reported by the current authors in a quantitative study using validated questionnaires (Makatsori & Miles, 2021). The aims of this study were to gain an understanding of adults’ experiences, feelings, and views of undergoing a food challenge test and the impact of this test using semi-structured interviews.

## Materials and methods

### Design

Qualitative research methods were chosen because they can provide in-depth information about people’s lived experiences. Semi-structured interviews were conducted to capture a range of perspectives and to allow for an in-depth understanding of the views, beliefs, and experiences of undergoing a food challenge test.

### Ethical considerations

For research projects involving the UK’s National Health Service, an application for ethical review is made on the Integrated Research Application System (IRAS) and a Research Ethics Committee (REC) is chosen to review the application. This study was allocated to, and approved by, the North East-Sunderland National Research Ethics Service (NRES) Committee (REC reference: 13/NE/0271). Research and Development approval was granted by the Royal Brompton and Harefield NHS Foundation Trust (2013AT007B).

Information sheets were sent to eligible participants to provide detailed information about the study. Potential participants were informed that participation was voluntary and that they had the right to withdraw at any time during the study, without affecting the standard of care they received. The researchers’ contact details were provided in case they had questions regarding the research. Participants were informed that the interviews would be recorded and transcribed. All participants who wished to participate in the study provided written informed consent prior to the interviews.

### Participants and recruitment

Participants were recruited from an Allergy Department in a hospital in the UK. The inclusion criteria were: age ≥ 18 years, diagnosis of IgE mediated allergy by allergy specialist (based on history & skin prick test + /- specific IgE tests + /- food challenge), previous food challenge or listed for food challenge test. Participants were required to have a good understanding and use of the English language in order to be able to express themselves during a discussion held in English.

A combination of convenience sampling and purposive sampling was used. Convenience sampling was used to identify adults who had previously undergone a food challenge test from a database (Participant information sheet (PIS) regarding the study was posted to them). In addition, individuals attending the clinic were approached and informed about the study and provided with written information. Purposive sampling ensured that the sample included variability in age, sex, food tested, and outcomes of food challenge tests (positive/negative).

Twenty-five individuals with food allergies were invited to participate. Twenty-four participants agreed to participate in this study, and 18 participants had undergone a food challenge and were therefore included in this study. The mean age was 34.7 years (SD = 11.8; range 20–56 years). 83% (*n* = 15) were female. Participants identified their ethnicity as White (61.1%, *n* = 11), Asian/Asian British (16.7%, *n* = 3), mixed/multiple ethnic groups (11.1%, *n* = 2) and Black/African/Caribbean/Black British (11.1%, *n* = 2). Length of time between the food challenge test and the interview varied with the majority of participants having had the challenge between 3−6 months prior to the interview, one 12 months prior, and three on the same day after the last challenge.

Foods that had previously caused symptoms included peanuts, tree nuts, shellfish, milk, egg, sesame, pine nuts, coconut, lupin, and fruits/vegetables (mango, avocado, nectarine, apple, and pear). Challenge tests were performed on different foods including tree nuts, peanuts, prawns, fruits/vegetables, eggs, and pine nuts. Participants had a negative food challenge for all the foods tested (*n* = 11), a positive reaction (*n* = 5), or tested positive for some foods and negative for others (*n* = 2). Twelve had developed symptoms to foods as adults, three in childhood, and three had symptoms of different foods in childhood, as well as experiencing symptoms to new foods as adults (see Table I).

**Table I.** Participant details.

Participant	Age	Gender	Onset	Foods involved in reactions	Food tested with challenge	Challenge outcome
P1	56	F	Αdult	Fruits & Vegetables (PFS), All nuts	Fruits, Vegetables, Nuts	Some positive, some negative
P2	27	F	Both	Fruits & Vegetables (PFS), All tree nuts	Brazil nuts	Negative
P3	27	F	Αdult	Mango, coconut, all nuts	Peanut & tree nuts	Negative
P4	21	F	Child	Peanut	Tree nuts	Negative
P5	32	F	Both	Cow’s milk, peanut & tree nuts	Egg	Negative
P6	37	F	Αdult	Shellfish	Prawns	Negative
P7	22	M	Child	Egg, cow’s milk, fish, peanut & tree nuts	Prawns	Negative
P8	56	F	Αdult	Tree nuts	Hazelnut	Positive
P10	26	F	Αdult	Avocado, spring onion	Onion, sushi	Positive
P11	40	F	Αdult	Peanut & tree nuts, fruits & vegetables	Nuts, pear	Positive
P12	36	F	Αdult	Peanut & tree nuts, Lipid transfer protein allergy, exercise	Exercise & nuts	Positive
P13	49	F	Αdult	Lupin PFS	Peanut	Negative
P15	23	F	Adult	Tree nuts, fruits (anaphylaxis to nectarine), vegetables	Vegetables, fruits	Some positive, some negative
P17	33	F	Αdult	Shellfish	Prawns	Positive
P18	46	M	Αdult	Peanut & tree nuts, fruits & vegetables	Nuts	Negative
P20	27	F	Both	Egg, peanut & tree nuts, vegetables & fruits (PFS), latex-food syndrome	Egg	Negative
P21	46	F	Αdult	Sesame, pine nuts, Peanut & tree nuts, fruits & vegetables	Nuts, pine nuts	Negative
P23	20	M	Child	Peanut & tree nuts	Tree nuts	Negative

PFS- Pollen Food Syndrome, F-Female, M-Male.

### Data collection

A topic guide was developed based on the aims of the research, literature review, and prior informal discussions with patients who had a history of food allergy or had previously undergone a food challenge test. The interview schedule was designed to ask participants about different aspects of living with food allergies, including symptoms, diagnosis, management, restrictions and effects on social and family life, their experience of undergoing a food challenge test, and the impact of the challenge outcome on their lives. The interviews were conducted in a private office at the hospital site or via telephone, depending on the participant’s choice. The interviews lasted up to a maximum of one hour.

Demographic data including sex, age, ethnicity and clinical characteristics, including type of food allergy and food tested with a challenge, were also collected through participant self-reporting and medical records. Following the initial transcription, the audio recording was again checked for accuracy, and identifying details (such as names of people and places) were removed.

### Data analysis

Thematic analysis was used for the purposes of data analysis to allow for an in-depth understanding of the views, beliefs, and experiences of affected individuals (Braun et al., 2022). Data analysis was assisted by NVivo qualitative data analysis software (QSR International, 2015).

Themes within the data were identified inductively, as the main aim of the thematic analysis was to provide a rich description of the entire dataset and to allow for the identification of novel findings regarding issues affecting the lives of food-allergic individuals and the impact of food challenges. The coding process was iterative, with constant data comparison to identify similarities and differences within and across interviews.

**Figure 1.**
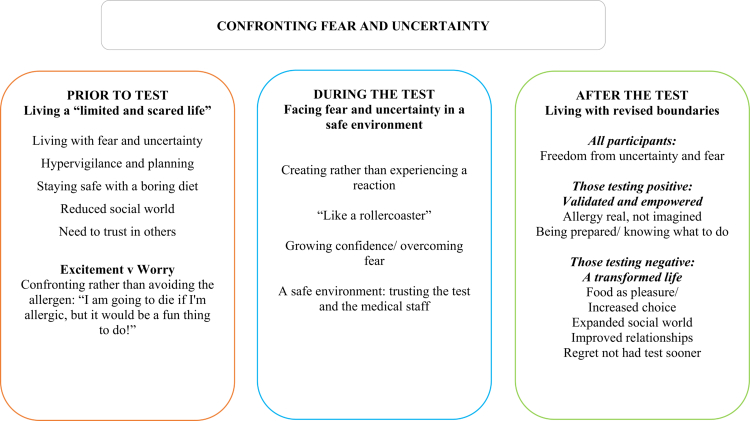
Diagram showing the relationships between themes and sub-themes.

Transcripts were read several times, and initial codes were generated across all transcripts. The codes and associated quotes were then reviewed for consistency across transcripts or overlap with other codes. Some initial codes were abandoned or merged at this stage because of overlap with others. The codes were then considered, and overarching themes and subthemes were developed from the coding groups, linking the data together. Themes were organised temporally, describing the experience of living with a food allergy before the food challenge test, the experience of the test itself, and life after the test (see Figure 1).

Coding was initially developed by MM who has clinical experience working with individuals with food allergies and conducting food challenges with reflexivity used to minimise potential bias. A reflective log was kept throughout data collection and analysis, open-ended and non-leading interview questions were used, and regular analytic discussions with AM took place. To enhance analytical rigour, a sample of transcripts was independently coded by AM with expertise in qualitative research methods but no clinical background in food allergy. Any discrepancies in coding or interpretation were discussed in detail between the researchers until consensus was reached. Any disagreements were resolved through reflexive discussion, ensuring that the themes remained grounded in the data and reflected the participants’ accounts.

## Results

The main themes identified were: **Prior** to the challenge test: Living a “limited and scared life” & Excitement vs worry for the test, **During** the test: Facing fear and uncertainty in a safe environment & “Like a rollercoaster” and **After** the test: Living with revised boundaries, Positive outcome (allergy confirmed)- Validated and empowered, Negative outcome (allergy excluded)- A transformed life.

### PRIOR to the challenge test: living a “limited and scared life”

Participants described **living in fear** because of the possibility of a reaction, particularly as a result of accidental exposure, and the **uncertainty** around whether they would react and how severe that reaction would be.

*I was constantly in fear, every part of my life, because I was constantly thinking nuts are everywhere.(I was) living a very limited and scared life …because I was so fearful of contamination, so my life for 12 years, was absolute hell.* (P21)

Living with food allergies constitutes a unique stressor due to unpredictability of potential reactions and their severity.

*I was worried about how reactions are unpredictable. That is not a nice thing to carry around, your psyche does not deal too well without certainty, to remove that uncertainty as far as is humanly possible it is really a positive thing.* (P6)

The need to avoid allergens resulted in **hypervigilance and the need for planning**.

*It is just the case of really checking everything and if in doubt don't eat it. You know. That is the only thing you can do really. (P8)*

*…if you're actually going out to buy something to eat at a cafe or from a bakery or whatever, you're having to think what's in it, what can you have and what's next to it and if they've used the same tongs for that as they used for the other. It has an impact every day on my life, every time I eat.* (P11)

In addition to being organised and pro-active regarding access to safe food such as cooking and carrying their own food, being prepared for any possible reactions including carrying emergency medications such as adrenaline auto-injectors when indicated, was another way that helped participants to feel more in control.

*I carry Epipens everywhere I go. I make sure that I am prepared more than anyone else.* (P18)

**Dietary restrictions** caused individuals to feel that their diets were monotonous and boring as well as being very limited and restrictive.

*Sometimes you feel that you end up with a boring meal, because you try to be safe all the time.* (P12)

*…all my life I take my packed lunch or going to a really boring Italian pizza restaurant, I'd really love to be able go to an Indian or a Thai or Chinese I cannot do that.* (P11)

All participants highlighted that living with food allergies lead to a **reduced social world**. It had significant social consequences, reducing their ability to participate in social activities as well as travel and do certain jobs. For example, one individual who worked as a chef and was unable to taste foods, and another who worked as a nanny who was asked to prepare foods she was allergic to. Individuals found it difficult to cope, as they did not know how to protect themselves, and their lives became increasingly restricted.

*I was too scared to go to a restaurant, to go out, it affects your social life…it was a nightmare. Going to restaurants with friends I just gave up.* (P1)

*it is very difficult even if you are going to a family…like Christmas for example they went to my cousin's place but I wouldn't go because the thing is you can't stop other people from having things in the house.* (P8)

Most participants mentioned that their friends and family were understanding of their allergies. For example:

*(they were) incredibly supportive, so it always tends to be a little bit easier (eating in) than restaurants and things…* (P11)

However, in some cases, relationships broke down as people stopped inviting them because of allergies.

*I got a real mixture, I even had people who stopped inviting me. and I lost contact with friends.* (P21)

Food allergies also affected relationships with partners. One participant felt that food allergies led to the breakup of her marriage.

*“my husband said I can’t continue with this, it’s so restrictive…because I was living in fear all the time that I might have an attack, …he said I can’t live like that I want to be able to travel, I want to be able to go to restaurants, and then even my ex-mother in law, she said how can you have a marriage when you can’t go to a restaurant?”* (P21)

In order to cope with the potential threats, individuals had to place their **trust in others**. Activities such as eating out and intimate contact with others comprised high-risk situations. Because they are not preparing their own food or in charge of what others eat, they depend on others’ understanding and knowledge of allergies to ensure that they were not exposed.

*It affected my social life in that I thought I would never be able to have another partner again, the trust issue… what if you kissed a guy who’s just had a snickers bar… kissing someone can put my life at risk and so a lot of the time is like I said, it’s like playing Russian roulette with your life.* (P1)

### Excitement versus worry

Participants were asked about their thoughts and feelings when they were initially advised in clinic that they should undergo a food challenge test to the suspect food and their emotions prior to having the test. Before having the food challenge, participants expressed that they were excited about having the test and felt it was a good opportunity to clarify their allergies and possibly help with expanding their diet.

*I was quite positive; I thought I'll definitely do this. I like eating a variety of food, so if I can incorporate more foods it is going only to be a benefit to me. (P23)*

*It sounded like a sensible thing to do because I need to have things in my diet that can be cooked quickly. (P5)*

*…to do so in a controlled environment it would seem a great opportunity so I was very glad.* (P6)

As the challenge test would involve **confronting rather than avoiding** the allergen as they had been doing, others were worried, apprehensive and nervous. However, they remained keen to find out whether they were allergic or not.

*I was a bit apprehensive but to be honest I wanted to know. Because peanut is in so many things.* (P13)

*Initial reaction was Oh My God! This is going to be interesting. But for me I'd rather know for sure what is causing it than not knowing.* (P17)

*I was a little bit worried. Obviously, I think inside you worry that something will happen.* (P4)

*The week of the test, I was stressed out, I’d been like panicking, because it’s just the whole thing that you’ve always been told that you’ll be dead if you eat nuts. and therefore to eat it, it just goes against all my kind of human nature.* (P2)

The contradiction between negative and positive perspectives for the test expressed by the same individual is best highlighted by the following quote:

*I'd say I was nervous, I thought I am going to die if I'm allergic, but it would be a fun thing to do! That was my first thought. Then I thought if I can find out that I can have tree nuts, then my son can also have them in his food.* (P4)

The strongly negative thought of the possibility of a severe reaction is contrasted immediately with a more positive thought and rational processing of the possible beneficial outcome.

When attending for a food challenge, individuals are informed by the clinical team what food they need to bring in with them for the test. Participants expressed how they found it strange that for the first time in their life instead of avoiding the food in question, they were actively seeking to purchase it to bring with them for the food challenge and the emotions this provoked.

*When I went shopping for the nuts it was a really strange experience because I was actively seeking out nuts in the shop instead of doing the opposite. So, having a shopping bag full of nuts was a strange experience.* (P20)

### DURING the test: facing fear and uncertainty in a safe environment

Having to eat a food as part of the food challenge test that has been avoided for years due to fear of allergic reactions provoked a mix of emotions. Individuals were aware that their minds might create symptoms similar to an allergic reaction due to anxiety and being acutely aware of every sensation in their body, trying to detect early signs of an allergic reaction.

*I was very conscious of the fact that sort of my mind was looking for something, I knew that any slight twitch in my mouth I was going to be oh God I am allergic* (P7)

*I felt a bit apprehensive, not sure what to expect because you know, how my body is going to react, my brain is going to react, psychologically more than anything else because I didn't have these nuts for a long long time, so I didn't know what to expect.* (P18)

However, by having this test in a controlled environment and with the help of the staff, they were able to differentiate between sensations like tingling, flushing, that can mimic an allergic reaction.

### “Like a rollercoaster”

The experience of undergoing the test and progressing through the increasing doses was described by one participant as follows:

“*like a rollercoaster, each little bit, like you would try something and you would be waiting for the reaction and getting all worked up waiting for the reaction, and then when the reaction didn’t come, you feel relieved and then the next bite, you wait again to see what will happen.”* (P1)

As the test progressed and participants did not experience any reactions, they felt more confident and reassured to continue with the test, have the next dose, and complete the test.

*Scary, it was really scary, but I felt quite safe here.It’s just at first, it’s a bit terrifying really. After, I’ve had half of a Brazil nut, I was fine. I was like, this is alright now.* (P2)

*As I went along and found that I wasn't having a reaction I felt reassured.* (P20)

Participants valued the simplicity of the test, that is, eating increasing amounts of the suspect food as this provided immediate and direct results and made the outcome of the test more believable.

*…unless you put something in your mouth and know for sure, then you’ll have that confidence that actually you’ll be fine. And you can eat it over and over again…Because I think if the approach hasn’t been so gentle, hasn’t been so supportive, hasn’t been so understanding I think it makes it so much harder because it’s a massive fear to overcome.* (P21)

*To do something like a food challenge is very concrete because although I was feeling ok this would give me the answer whether I am not allergic to shellfish, you still do not want to mess around with your health, to do so in a controlled environment it would seem a great opportunity, so I was very glad.* (P6)

They felt safe and expressed their gratitude to the healthcare staff that performed the food challenges and conveyed their satisfaction with the care they had received.

*I felt really safe. There were members of staff observing you all the time, making sure you don't react.* (P4)

For those testing positive, symptoms varied from skin rashes or swelling to anaphylaxis during the test. Despite experiencing a reaction, they still felt that the experience of undergoing the test was worthwhile and useful.

*I know that I am allergic to food and I know I can have anaphylaxis and that's fine, so there is always a risk that that was going to happen…in a way it was positive because it meant actually that doctors can make the right diagnosis.* (P11)

### AFTER the test - living with revised boundaries

There was consensus among participants that having a food challenge test made a significant difference in their lives. Immediately following the challenges, individuals expressed that they were glad to have completed the test and were excited for the future.

*I was excited. I wanted to start a diet and a lot of the stuff had oats and nuts. I can actually have a healthier diet now. (P4)*

*I was glad I've done it. I've done it now, I can be a bit more relaxed, I tried things now (P18)*

The common theme for all participants was ***Freedom from uncertainty and fear.*** Undertaking a food challenge test provided clarity as to whether an individual was allergic to a specific food, and cleared any ambiguity regarding equivocal skin prick and blood tests. Participants found eliminating this uncertainty and fear to be beneficial, even when they still tested positive for some allergens. They were aware, that if they did not have the opportunity to have a challenge test, their lives would have continued as before:

*Without the challenges my life would be continuing in fear… It reassures you because nothing else would…So, I can’t thank the staff enough, they **transformed** my life, miracle.* (P21)

Testing provided reassurance and clarity as to what foods they were able to eat or needed to continue to avoid.

*I was really nervous about going to restaurants and I didn't know what foods to avoid because it wasn't like a food, it was a topping that made me sick, which I didn't understand but now I know and it's fine… This has helped me a lot, now actually I know what not to eat and what I can eat.* (P10)

### Positive test - validated and empowered

Individuals who had a positive outcome i.e., reacted to the food tested, were relieved to identify the cause of their symptoms. It enabled them to confirm their suspicions that they are allergic to that food.

They felt validated that the allergy was real, not imagined. As this participant explained, individuals with unconfirmed allergy may start to question themselves, wondering if they are overreacting. They worry that others may think that they are exaggerating or are ‘being fussy’. A confirmed diagnosis removes these doubts. It validates that the experience is real, provides a stronger sense of identity, fosters a better sense of self-acceptance.

*At least now this is confirmed other people including other doctors can now believe me. The challenges have been a lot of help to confirm my suspicions, otherwise you just feel as if you're making a fuss.* (P12)

**Being prepared/knowing what to do-** By confirming what they could and could not eat, the challenge test helped to empower them to focus on coping strategies including ensuring that they are well-prepared to treat any reactions, carrying adrenaline auto-injectors if indicated and having an anaphylaxis action plan. Furthermore, it emphasised the need to implement measures to minimise risk e.g., when travelling carrying a list of their allergies translated in other languages, informing others of their allergies.

*I am completely happy to know what is causing it, because I can manage it a lot better now.* (P17)

*Travelling abroad, what I tend to do is I tend to get a little statement listing all the foods I am allergic to. So, I’ve got a little piece of paper so even if they don't understand me I can show it to them.* (P12)

Two participants, who experienced anaphylaxis during the challenge, still felt that the experience and undergoing the test was useful and did not regret having the test.

*Well, there are two ways of looking at this. Look, I had such a bad reaction, if I had known this was going to happen I wouldn't have had it done. But on the other hand, I now know that this is definitely what it is and through that really it kind of led to other things really. There are two sides of the coin there.* (P8)

*…Because I knew what was happening, I know how it feels and I knew that the doctors were going to make things all right and that it will be OK, it was fine, it didn't bother me.* (P11)

The experience of having a reaction in a controlled environment, provided reinforcement of how to best manage any reactions, including early administration of adrenaline. It helped to build confidence by teaching individuals to recognise symptoms and act quickly and built psychological resilience. It helped them mentally prepare for future allergic incidents and feel that they have the tools to manage the situation and reduce the fear of the unknown.

When a health condition is recognised and confirmed, it is also easier for others to understand and support that person. Family members, friends and co-workers can offer help or emotional support if they know what the condition is. This strengthens relationships and can help people feel less isolated or misunderstood. People with confirmed allergies felt empowered to implement changes in their workplace. For example, one individual who worked as a chef said:

*I have to try different foods, taste them, obviously I am conscious what is in the foods but I have a team at work, I have another chef who tastes for me now.* (P18)

### Negative test outcome - a transformed life

Prior to the food challenges, individuals were following ‘boring’, restricted diets and were seeing food as a potential ‘danger’. The food challenges enabled them to reintroduce foods that they tested negative to as well as expand and improve their diet.

*It opened up a whole new thing for me to be able to eat stuff like that, which was great. It was really positive and made a big difference, you know you get bored, the thing is when you cut out so many foods you get so bored, so actually to have the opportunity to refine that list, and actually find then something positive to have it was really good.* (P11)

#### Food as pleasure/increased choice

Participants were very excited that they were able to re-introduce foods that they were avoiding in some cases for many years. They were pleased that they could enjoy different foods again but more importantly they found food became a source of pleasure rather than fear.

*You won’t believe! I sat there and I was eating almonds, cashew nuts and pesto I absolutely love pesto, I was eating Nutella by the bucket… I am able to indulge a lot more. This summer has been amazing; I’ve been enjoying such fresh fruit, melon, water melon, things that I would just dream at just looking at them. It’s been such a delight to be able to enjoy, the complete joy of life.* (P21)

*I will be having a jar of nuts in my kitchen of each type, I will be including it in all my salads and foods. I think I will be including it in EVERYTHING.* (P2)

Some individuals who had avoided the food tested since childhood had no previous experience of how this food even tasted or in what dishes it was used but they were still excited about the increased choices they had now.

*I’ve never had them (prawns) before, it will take me a while to work out how to eat them and some recipes, that's the thing I need to find. What do I eat prawns with, what do I have, when do I eat oysters, I just don't know but it's exciting.* (P7)

Re-introducing a food that someone has been avoiding for years due to fear of reactions, takes a lot of courage because of the preconditioning and habit of avoidance developed over time. The brain becomes conditioned to associate that food with potential danger, leading to heightened fear and anxiety. Understandably, initially in some cases participants were still cautious to start with food re-introduction.

*In retrospect it has taken some months, it’s really only in the last few months that I am not always looking at labels, I’m confident enough to try something that previously thought I could have a reaction to*. (P1)

Participants found referring back to their experience of eating food during the challenge test useful to reassure themselves if they felt that they were experiencing symptoms when they tried the food again on their own.

*When I was eating the nectarines I was starting to get that sensation and I’d start to get a bit panicky, I’d think No! you’ve been through the food challenges you know it’s ok to eat that, you know it’s just a sensation on your tongue, it’s not a reaction, I think in that respect it gave me more confidence and the ability to be able to deal with it.* (P1)

Participants who had some negative tests, but still had allergies to other foods, found the challenges tests were still of benefit as they now had an increased choice and new alternatives that they can introduce.

*Now I can have egg, it will make things so much easier for me, because I mean you can boil an egg in 10 minutes that is so quick.* (P5)

#### Expanded social world

People were able to re-evaluate the impact food allergies exerted on their social lives including eating out, shopping, travelling and were able to experience these differently with less worry. This was the case even when they remained allergic to some food groups. The newly acquired knowledge enabled them to have new experiences and expand their social world.

*And I’m so grateful, you can’t imagine, I’ve been away, because before I wouldn’t be able to go and stay in a hotel…I am delighted that I just got my life back. Now I go to hotels, and I go to restaurants and I know exactly what to avoid and you can have a complete life now without worrying because you have choices now. Before I wouldn’t have much of a choice and I am absolutely delighted* (P21)

*It changes things. My job involves a lot of travelling, so I have to go to China, and something like this means I’ll be able to go… It really does have a massive impact for me because it just takes away the worry.* (P2)

Furthermore, it enabled them to do things like free up the way they shopped, all tasks that individuals without food allergies take for granted.

*I have tomato all the time, to be able to have that it’s incredible, to be able to walk into a supermarket and pick up a banana and eat it without having to panic, to be able to pick up a sushi packet and not have to worry that the poppy seeds and sesame seeds are going to put me in hospital.* (P1)

#### Improved relationships

In addition, they noticed an improvement not only in their lives but also in the lives of family members such as partners and children as well as friends. As their dietary and social restrictions were also having an impact on others, following a negative challenge outcome, family members were able to eat the foods previously avoided due to the risk to the allergic individual and social events were also less affected.

*Also, my family get really annoyed when we go out for a meal- I always have to ask if there are any nuts in there. They’re like, oh, here she goes again…. my life will change. It will mean I will be able to go to a restaurant and choose anything, I won’t have to worry about what I’m choosing. And that is SO good.* (P2)

*…and to my children who are 6 and nearly 4 who've never seen me eat nuts for them it was like a bit are you sure mummy?? And yeah actually I can now, to them they didn't even knew these things existed, so now my sons have tried them and my 6-year-old loved the cashew nuts, you know because I was allergic I never had it in the house, so it prevented them accessing stuff as well.* (P11)

#### Regret not had test earlier

The only regret they had was that they did not have the food challenges earlier, and had very limited diets for years before testing. They felt that earlier testing, could have prevented unnecessary restrictions.

*The only thing I regret is that I didn't do it a year ago or two years ago, I waited 6 years from the last one when I was signed off by the children's ward. I wish I'd done it all sooner really and I look forward to eating shellfish.* (P7)

## Discussion

This study explored the adults’ perspective of undergoing a food challenge test to investigate food allergies, and the impact of this test on their health-related quality of life. The themes highlighted that, prior to having a food challenge, participants lived in fear and uncertainty. They were following very restricted diets, which impacted their social lives and relationships with others. While the food challenge test involved confronting their fear, participants reported being able to do this in a safe clinical environment. Following the food challenge, participants reported improvements in their quality of life, with many individuals describing this as a transformation. For participants who tested positive, there were feelings of relief to know the precise causes of their symptoms and validation that they were allergic. For those testing negative, the results represented freedom from fear and unnecessary restrictions.

The present study shows that living with food allergies negatively impacts affected adults, and generates considerable fear and uncertainty. This is in agreement with recent qualitative research showing that adults with food allergies experience a constant psychological burden, including anxiety, stress, and social limitations (Roleston et al., 2025). Moreover, individuals with multiple food allergies are particularly affected, facing heightened stress regarding food safety, allergen avoidance, and restrictions on freedom (Ciaccio et al., 2024).

According to the Uncertainty in Illness Theory, uncertainty is generated when aspects of illness or treatment possess the characteristics of inconsistency, randomness, complexity, unpredictability and lack of information in situations of importance to the individual (Mishel, 1981; Mishel, 1988). Uncertainty about a possible future threat, as is the case in food allergies, disrupts the individual’s ability to avoid it or to mitigate its negative impact and can result in anxiety, worry, and perceptions of vulnerability (Hillen et al., 2017, Hsu et al., 2005; Lang et al., 2000; Rosen & Donley, 2006). The findings of our study support the view that by confirming the presence or absence of an allergy using the food challenge test, the fear and uncertainty around food can be minimised or even eliminated. Exposure to a feared stimulus in a controlled environment was liberating for many participants. This finding can be interpreted through Bandura’s self-efficacy theory, which emphasises the importance of perceived capability in managing potentially threatening situations (Bandura, 1977; Bandura, 1997). Oral food challenges can be viewed as experiences facilitating mastery, enabling participants to directly test their ability to tolerate or manage exposure to specific foods in a safe environment.

All participants in our study reported significant benefits from undergoing the oral food challenge, as described in the theme “living with revised boundaries”. Consistent with self-efficacy theory, participants who tested negative experienced increased confidence and reassurance, enabling them to later reintroduce the food into their own and their family’s diet, and to experience it as pleasurable rather than threatening. In the case of individuals testing positive, the test revealed which allergens they needed to avoid and reduced uncertainty, enabling them to cope more effectively with their allergies. Regardless of the test outcome, participants reported being able to take part in more social activities, such as eating out and travelling, as a result of the certainty provided by the test results. In addition to being a diagnostic tool, a food challenge test thus has a therapeutic effect as an uncertainty management intervention.

Of note, despite some individuals experiencing severe reactions during the test, the validation of their condition, the practical learning and increased psychological resilience gained, made the difficult experience worthwhile for them. This is in keeping with exposure theory- encountering a feared reaction in a safe, supervised environment reduces fear by disconfirming catastrophic expectations and demonstrating that prompt intervention is effective (Craske et al., 2014). Also in line with cognitive appraisal theory, post-challenge individuals were able to re-evaluate reactions as manageable, as they had experienced what the symptoms were and had increased confidence that treatment would be effective. This cognitive reappraisal can lead to improved coping and reduced emotional distress (Lazarus & Folkman, 1984).

While previous qualitative research has examined maternal and adolescent experiences of undergoing oral food challenges rather than adults, the feeling of fear, evident in our first two themes, is also dominant in these studies (Correa et al., 2022; Strinnholm et al., 2010; Strinnholm et al., 2017). For example, the theme *“living with fear of the unknown”* describes mothers’ fears, both during their child’s negative double-blind, placebo-controlled food challenge (DBPCFC) and after the food challenge when reintroducing foods to which their child had tested negative (Strinnholm et al., 2010). In the study by Correa et al., (2022), adolescents undergoing oral food challenges reported a mix of fear and anxiety, relief, and empowerment.

While participants in our study stated that they reintroduced the food for which they tested negative during the challenge test, some reported hesitancy in doing so. This is consistent with findings from other research (Strinnholm et al., 2010; Strinnholm et al., 2017). In a study of adolescents who underwent DBPCFCs to milk, egg, or cod, some participants who tested negative continued ‘old habits’, either because they did not like the food they had avoided for years or because they still felt unable to eat it (Strinnholm et al., 2017). It is important to note that, in most cases, individuals spend years prior to the test avoiding that specific food, becoming preconditioned to avoidance. This long-term avoidance can make reintroduction difficult despite reassurance from a negative test. This can be understood through classical conditioning and avoidance learning theories, which support how repeated avoidance reinforces associations between the food and perceived danger, maintaining fear and anxiety over time (LeDoux et al., 2022; Rachman, 1991). Current practice is that individuals testing negative are discharged from clinical outpatient care; however, some form of follow-up, such as a telephone consultation by a dietitian or clinician, may help individuals feel more confident and supported, especially when reintroducing multiple foods.

A key strength of this study was its focus on adults, contributing to the limited research available in this age group. This also contributed to identification of specific issues faced by adults, for example, the impact on the affected individuals’ children, partners, friends and work related issues. In addition, examining open food challenges provided a more practical perspective compared with DBPCFCs, making the findings more applicable to routine clinical practice. Another strength was the inclusion of participants from a range of age groups, ethnicities, socioeconomic backgrounds, food allergies, and reaction severities. Participants underwent challenges to different foods and had varying test outcomes (positive or negative). This heterogeneity allowed for a broader assessment of the difficulties faced by individuals with food allergy and the impact of food challenges, capturing both shared and divergent perspectives and providing rich data for analysis.

Several limitations should be noted. Although both female and male participants were included, the majority were female, reflecting evidence that food allergies are more prevalent among women than men (Afify & Pali-Schöll, 2017) and that women are more likely to participate in research and seek help for health-related issues (Addis & Mahalik, 2003; Galdas et al., 2005; Patel et al., 2003). However, gender differences may exist in how individuals perceive and cope with food allergies and diagnostic testing. Previous research suggests complex interactions between biological sex, gender, and health, which may contribute to sex- and gender-related differences in the biopsychosocial manifestations of food allergy (DunnGalvin et al., 2006). Future research should aim to include a greater proportion of male participants.

Another limitation is that this was a single-centre study conducted within a specialist allergy department. A multi-centre design may have enhanced the credibility of the findings and reduced the likelihood of bias arising from care-related factors that may have positively influenced participants’ experiences. Furthermore, it is important to note that this study included only adults who completed an oral food challenge, excluding those never referred, too anxious to proceed, or without access to such a service, and thus only reflects the experiences of these treated and challenged adults. Limiting participation to English-speaking individuals may have introduced selection bias, under-representing patients with limited English proficiency or lower health literacy. In addition, most participants were interviewed three to six months post-food challenge. While this delay may have introduced recall bias, it allowed for an assessment of the longer term benefits of the oral food challenge, and our results are consistent with a previous prospective quantitative study showing improved quality of life three months after testing, compared to baseline levels (Makatsori & Miles, 2021). Nevertheless future qualitative research could use a longitudinal design to better understand how time affects people’s reported experience of undergoing a food challenge.

The importance of providing a safe environment was evident in this study. According to trust theory, patients rely on healthcare professionals’ competence and a safe clinical environment when perceived risk is high, as is the case in food challenges (Hall et al., 2001). Trust in the process can be enhanced by ensuring that patients have met the clinical team carrying out the food challenge test before it takes place. Good staff communication also assisted in providing emotional reassurance to participants as well as acting as a source of distraction and motivation to complete the test. It is important to consider this when delivering food challenge services and tailoring staff interactions accordingly, based on each person’s needs.

The participants’ experiences highlighted that a significant length of time passes between an individual experiencing a possible allergic reaction and undergoing a food challenge test. In previous quantitative research, the average time varied between 8.6 years and 12.5 years (Makatsori & Miles, 2021). This may be due to delays in referring to specialist allergy services and/or a lack of service provision for food challenges for adults in the UK. It would be of interest to explore whether individuals who wait longer for the test find it more difficult to change their avoidance behaviours and reintroduce the food if they test negative.

Overall, the results of this study highlight the benefits of food challenge tests, but also illustrate the need for further research into the availability, uptake and consequences of undergoing such tests in the adult population.

## Conclusion

This study explored the experience and impact of undergoing a food challenge test in adults and identified themes leading to improved quality of life. Individuals who have experienced possible allergic reactions are left to live their lives burdened by uncertainty and fear, dramatically restricting enjoyment of food and participation in social activities. Undergoing a food challenge test enabled participants to confront and resolve fear and uncertainty around having an allergy, and improved their quality of life by releasing people from previous unnecessary constraints among those testing negative, and providing clear boundaries to those testing positive by confirming which foods they were allergic to. In addition to being a diagnostic tool, a food challenge test also has a therapeutic effect as an uncertainty management intervention. The findings of this study support the need for continued provision of adult food allergy services and food challenge testing and consideration of more widespread access to these.

## Data Availability

Data will be made available upon reasonable request.
